# Case Report: Inversion of *LMX1B* - A Novel Cause of Nail-Patella Syndrome in a Swedish Family and a Longtime Follow-Up

**DOI:** 10.3389/fendo.2022.862908

**Published:** 2022-06-13

**Authors:** Hillevi Lindelöf, Eva Horemuzova, Ulrika Voss, Ann Nordgren, Giedre Grigelioniene, Anna Hammarsjö

**Affiliations:** ^1^ Department of Molecular Medicine and Surgery, Center for Molecular Medicine, Karolinska Institutet, Stockholm, Sweden; ^2^ Department of Clinical Genetics, Karolinska University Hospital, Stockholm, Sweden; ^3^ Department of Pediatric Radiology, Karolinska University Hospital, Stockholm, Sweden

**Keywords:** nail-patella syndrome, LMX1B, inversion, skeletal dysplasia, next generation sequencing, case report

## Abstract

Nail-patella syndrome (NPS, OMIM #161200) is a rare autosomal dominant disorder with symptoms from many different parts of the body, including nails, knees, elbows, pelvis, kidneys and eyes. It is caused by truncating variants in the *LMX1B* gene, which encodes a transcription factor with important roles during embryonic development, including dorsoventral patterning of the limbs. To our knowledge, inversions disrupting the *LMX1B* gene have not been reported. Here, we report a family with an inversion disrupting the *LMX1B* gene in five affected family members with mild but variable clinical features of NPS. Our finding demonstrates that genomic rearrangements must be considered a possible cause of NPS.

## Introduction

Nail-patella syndrome (NPS, MIM#161200) is a rare autosomal dominant disorder that affects many different parts of the body, including nails, knees, elbows, pelvis, kidneys and eyes. The reported incidence is 1 in 50 000 but could be higher due to individuals with a mild phenotype that remain undiagnosed (1). The majority present with nail abnormalities, including split or ridged, underdeveloped or absent nails (1, 2) with more severe nail involvement on digits 1-3. The skeletal abnormalities include elbow deformities and radial head dislocation, patellar hypoplasia or aplasia and horn-like outgrowths of the pelvis (iliac horns) (1). Extraskeletal symptoms include renal, ocular and neurological symptoms. Renal involvement first manifests as proteinuria with or without hematuria and occurs in 30-50% of the cases and progresses to end-stage renal disease in 5% (1, 2). The molecular mechanisms underlying the nephropathy remain unclear, but electron microscopy shows thickening of the glomerular basement membrane and loss of podocyte foot processes (2). There is also an increased risk of glaucoma and sensorineural hearing impairment in patients with NPS (1).

NPS is caused by heterozygous mutations in the *LMX1B* gene, encoding a LIM-homeodomain transcription factor with an important role during embryonic development (3). *LMX1B* is involved in dorsoventral patterning of the limbs, fusion of podocytes at the kidney’s glomerular basement membrane, the development of the anterior portions of the eyes and differentiation of specific neurons in the central nervous system. The LMX1B protein comprises one highly conserved DNA-binding homeodomain and two protein-binding LIM domains composed of two zinc fingers (LIM-A and LIM-B) (4). Most disease-causing variants are located in these domains and result in a non-functional LMX1B protein or reduced DNA-binding ability (5).

## Patient Information

**Figure 1:**
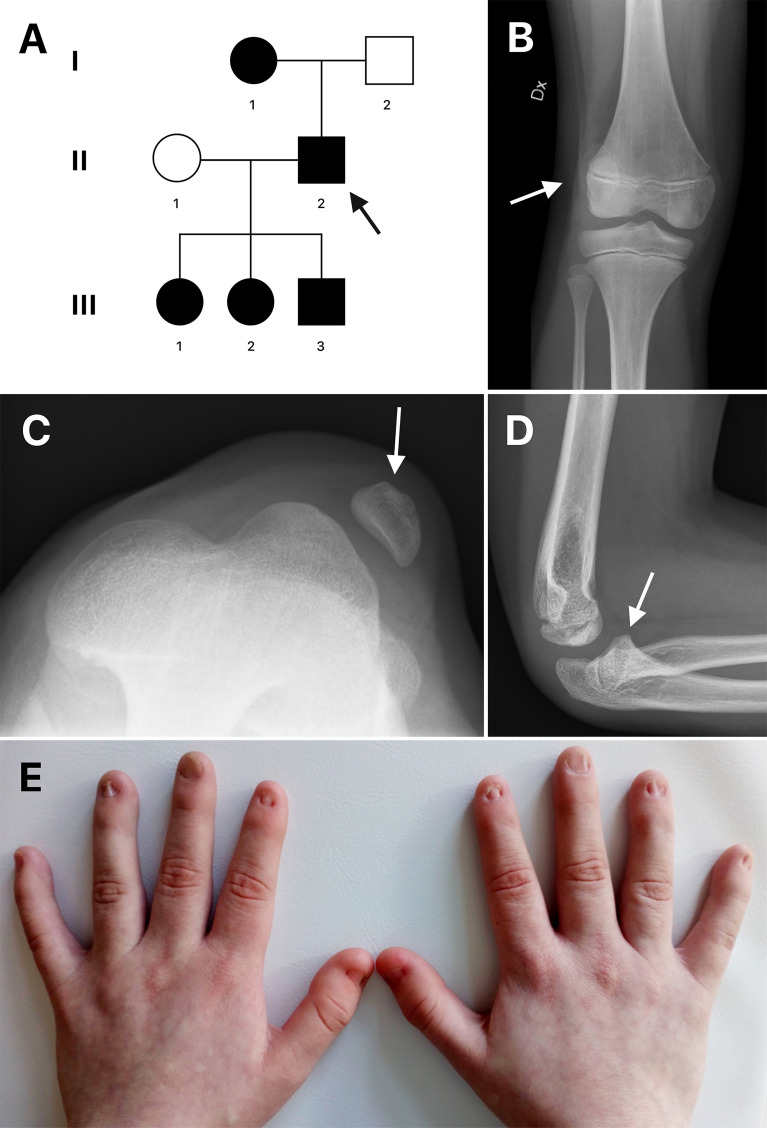
**(A)** Pedigree of the family. Circles represent females and squares represent males. An arrow indicates the proband. Filled shapes represent affected individuals. **(B, C)** Radiographs of the right and left knee of patient III:1 showing laterally dislocated hypoplastic patella (arrows). **(D)** Radiograph of the left elbow of patient III:1 shows posteriorly dislocated radial head (arrow). **(E)** Dysplastic nails of patient III:2.

The studied family is of Swedish descent and includes a 50-year-old man (proband), his mother and three children (**Figure 1A**). We present a long-time clinical follow-up that spans nine years. The proband was diagnosed with NPS in his childhood based on characteristic clinical findings of dystrophic nails combined with an absent left patella. According to the proband, his mother had the same dystrophic nails, and he recognized similar symptoms of NPS in his three children. In 2011, the father and his children aged 5, 6 and 8 years presented at the skeletal dysplasia team at Karolinska University Hospital for evaluation and genetic testing. The children were followed-up annually with clinical examination, urine analysis and eye examination. The clinical findings of the affected individuals are summarized in **Table 1** and **Figure 1**. All had short stature of varying degree, dystrophic nails, elbow anomalies except III:2 and hypoplastic or absent patella. None had iliac horns, nephropathy or ocular involvement. During follow-up, two of the children, the oldest daughter and the son, required patellar stabilization surgery due to recurrent dislocations.

**Table 1:** Summary of clinical characteristics.

Patient	Sex	Age (year)	Nail dysplasia	Patellar anomaly	Elbow anomaly	Iliac horns	Nephropathy	Ocular involvement	Height (cm)	Height (z-score*)
I:1	F	75	+	+	+	–	–	–	142	-3,2
II:2	M	50	+	+	+	–	–	–	163	-1,9
III:1	F	17	+	+	+	–	–	–	154	-1,4
III:2	F	15	+	+	–	–	–	–	151	-1,7
III:3	M	14	+	+	+	–	–	–	157	-0,8

*Z-score is calculated by WHO child growth standards (6).

## Clinical Findings

The proband (II:2) had patellar hypoplasia on the right knee and dislocated radial heads on both elbows. During his childhood he was overall healthy except for limited extension of his forearm. He was regularly examined with urine analysis and eye examination with no signs of hematuria or kidney involvement.

The eldest daughter (III:1) had hypoplastic patellae (**Figures 1B, C**) with reoccurring dislocations on both sides. She required bilateral patellar stabilization surgery at the age of 11. Her radial heads were dysplastic due to chronic dislocation on both sides, posterior on the left side and anterior on the right side. The left elbow had an extension defect of approximately 20 degrees (**Figure 1D**).

His second daughter (III:2) had a milder phenotype. She had a slightly underdeveloped right patella, bipartite left, and no dislocations. Her fingernails were dystrophic (**Figure 1E**).

His son (III:3) had hypoplastic patellae bilaterally with reoccurring right knee dislocations, requiring patellar stabilization surgery at 13 years. The elbows had a valgus deformity and the radial heads were dislocated.

## Diagnostic Assessment

**Figure 2:**
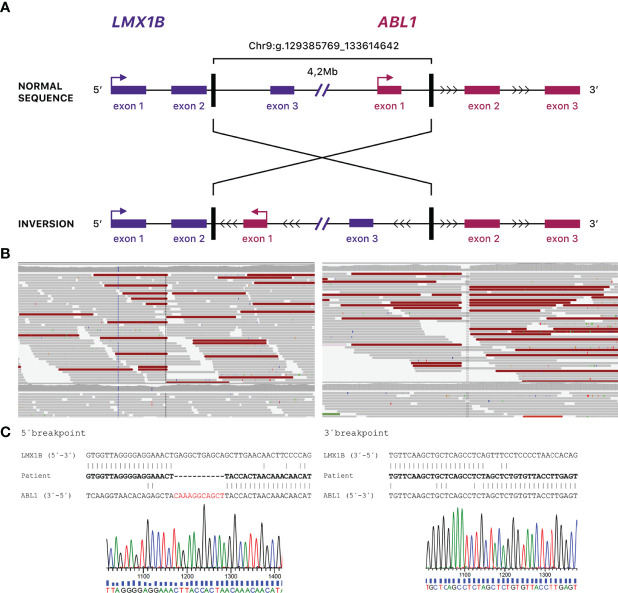
**(A)** Schematic depiction of the inverted region. Vertical lines show the breakpoints. Note that the figure is not drawn to scale. **(B)** Whole genome sequencing data of the breakpoints of the inversion. **(C)** Schematic presentation of the breakpoint junction between intron 2 in *LMX1B* and intron 1 in *ABL1* and sequence traces over the breakpoint junctions.

The study was approved by the regional ethical review board in Stockholm, Sweden (2014/983-31/1), and written informed consent from the patients was obtained. Sanger sequencing of *LMX1B* was performed for patients II:2 and III:2. However, the analysis failed to detect any pathogenic *LMX1B* variants. Whole genome sequencing of patient III:1 was performed using a PCR-free paired-end protocol (Illumina TruSeq DNA PCR-free) using a HiSeq X Ten system (Ilumina). All called and annotated variants were given a prioritization score by applying a rank model and uploaded into Scout, an interface for variant analysis, as previously described (7). The analysis revealed an inversion stretching approximately 4,2 Mb on chromosome 9, NC_00009.12:g.129385769_133614642, disrupting *LMX1B* and *ABL1*. A small deletion of eleven nucleotides was found at the 3’breakpoint (**Figure 2**). Sanger sequencing over the breakpoints confirmed these results and showed that all affected family members were carriers of the inversion. The predicted consequence of the inversion is disruption of *LMX1B* in intron 2, which would lead to a hybrid transcript including exons from both *LMX1B* and *ABL1*.

## Discussion

Most NPS cases are caused by heterozygous loss-of-function variants in the *LMX1B* gene. More than 170 pathogenic variants are known, and most of them are found in the LIM domains (1, 3, 4, 8–10). The majority (88%) of the cases are inherited from an affected parent, and 12% are *de novo* (1). Symptoms may vary considerably from mild to severe, even amongst family members. Renal involvement and progression to end-stage renal failure is the most severe complication associated with NPS. Here we report the first case of NPS caused by an inversion disrupting the *LMX1B* in five affected family members, all had variable symptoms of NPS. The molecular consequence of the inversion, if translated, would be a potential hybrid transcript, resulting in a non-functional protein with a frameshift and a premature stop codon after eight amino acids. This predicted truncation would lead to a lack of critical functional domains of *LMX1B*, including LIM-B and homeodomain. It was not possible to analyze this transcript in our family due to low expression of *LMX1B* in blood and skin. Aberrant recognition of exon-intron junction sites is another possible consequence since the inversion is large. The *ABL1* gene is a proto-oncogene known as part of the fusion gene BCR-ABL1 in the Philadelphia chromosome of leukemia cells. Missense mutations in the *ABL1* gene have been described in six individuals within four families with skeletal malformations and congenital heart defects (CHDSKM, MIM #617602) (11). However, only missense mutations are described in *ABL1*, and the disease mechanism is therefore suggested to be gain-of-function. In contrast, as this rearrangement inverts the entire first exon of *ABL1* and its promotor, it will not make a functional protein. In the gnomAD database, loss of function variants in *ABL1* are rare, but haploinsufficiency of *ABL1* is not implicated as a disease mechanism. Thus we do not expect any clinical symptoms of CHDSKM for our patients. The inversion comprises several genes, and their change in direction or disruption of a topologically associating domain may potentially affect the expression. No other genes apart from *LMX1B* and *ABL1* are disrupted by the inversion, in close proximity to the breakpoint region, or are known to cause an autosomal dominant disease. In conclusion, our findings expand the molecular spectrum of NPS, indicating that genomic rearrangements should be considered a possible cause of NPS in patients where standard genetic investigations fail to detect any pathogenic variants in *LMX1B*.

## Data Availability Statement

The datasets for this article are not publicly available due to concerns regarding participant/patient anonymity. Requests to access the datasets should be directed to the corresponding author.

## Ethics Statement

The studies involving human participants were reviewed and approved by Regional ethical review board in Stockholm, Sweden. Written informed consent to participate in this study was provided by the participants’ legal guardian/next of kin. Written informed consent was obtained from the individual(s), and minor(s)’ legal guardian/next of kin, for the publication of any potentially identifiable images or data included in this article.

## Author Contributions

Conception and design of the work: AH and GG. Data collection: HL, EH, and GG. Data analysis and interpretation: HL, AH, AN, and GG. Drafting the article: HL. All authors reviewed the results and approved the final version of the manuscript.

## Funding

AH: The Swedish Rare Diseases Research foundation (Sällsyntafonden), Sällskapet Barnavård and Karolinska Institutet. HL: Karolinska Institutet. GG and AN: Grants from Region Stockholm (20180131 and 20200500), from Swedish Research Council (2018-03046), and Karolinska Institutet. GG also has grants from Promobilia Foundation and Frimurare Barnhuset foundation in Stockholm and AN from the Hållsten Research foundation.

## Conflict of Interest

The authors declare that the research was conducted in the absence of any commercial or financial relationships that could be construed as a potential conflict of interest.

## Publisher’s Note

All claims expressed in this article are solely those of the authors and do not necessarily represent those of their affiliated organizations, or those of the publisher, the editors and the reviewers. Any product that may be evaluated in this article, or claim that may be made by its manufacturer, is not guaranteed or endorsed by the publisher.

## References

[1] Sweeney E Fryer A Mountford R Green A McIntosh I. Nail Patella Syndrome: A Review of the Phenotype Aided by Developmental Biology. J Med Genet (2003) 40(3):153–62. doi: 10.1136/jmg.40.3.153 PMC1735400 12624132

[2] Bongers EM Huysmans FT Levtchenko E de Rooy JW Blickman JG Admiraal RJ. Genotype-Phenotype Studies in Nail-Patella Syndrome Show That LMX1B Mutation Location is Involved in the Risk of Developing Nephropathy. Eur J Hum Genet (2005) 13(8):935–46. doi: 10.1038/sj.ejhg.5201446 15928687

[3] McIntosh I Dunston JA Liu L Hoover-Fong JE Sweeney E. Nail Patella Syndrome Revisited: 50 Years After Linkage. Ann Hum Genet (2005) 69(Pt 4):349–63. doi: 10.1111/j.1529-8817.2005.00191.x 15996164

[4] Dunston JA Hamlington JD Zaveri J Sweeney E Sibbring J Tran C. The Human LMX1B Gene: Transcription Unit, Promoter, and Pathogenic Mutations. Genomics (2004) 84(3):565–76. doi: 10.1016/j.ygeno.2004.06.002 15498463

[5] Marini M Bocciardi R Gimelli S Di Duca M Divizia MT Baban A. A Spectrum of LMX1B Mutations in Nail-Patella Syndrome: New Point Mutations, Deletion, and Evidence of Mosaicism in Unaffected Parents. Genet Med (2010) 12(7):431–9. doi: 10.1097/GIM.0b013e3181e21afa 20531206

[6] Grummer-Strawn LM Reinold C Krebs NF Centers for Disease C Prevention. Use of World Health Organization and CDC Growth Charts for Children Aged 0-59 Months in the United States. MMWR Recomm Rep (2010) 59(RR-9):1–15. 20829749

[7] Stranneheim H Lagerstedt-Robinson K Magnusson M Kvarnung M Nilsson D Lesko N. Integration of Whole Genome Sequencing Into a Healthcare Setting: High Diagnostic Rates Across Multiple Clinical Entities in 3219 Rare Disease Patients. Genome Med (2021) 13(1):40. doi: 10.1186/s13073-021-00855-5 33726816 PMC7968334

[8] Bongers EM Gubler MC Knoers NV. Nail-Patella Syndrome. Overview on Clinical and Molecular Findings. Pediatr Nephrol (2002) 17(9):703–12. doi: 10.1007/s00467-002-0911-5 12215822

[9] Ghoumid J Petit F Holder-Espinasse M Jourdain AS Guerra J Dieux-Coeslier A. Nail-Patella Syndrome: Clinical and Molecular Data in 55 Families Raising the Hypothesis of a Genetic Heterogeneity. Eur J Hum Genet (2016) 24(1):44–50. doi: 10.1038/ejhg.2015.77 25898926 PMC4795216

[10] Harita Y Urae S Akashio R Isojima T Miura K Yamada T. Clinical and Genetic Characterization of Nephropathy in Patients With Nail-Patella Syndrome. Eur J Hum Genet (2020) 28(10):1414–21. doi: 10.1038/s41431-020-0655-3 PMC7608088 32457516

[11] Wang X Charng WL Chen CA Rosenfeld JA Al Shamsi A Al-Gazali L. Germline Mutations in ABL1 Cause an Autosomal Dominant Syndrome Characterized by Congenital Heart Defects and Skeletal Malformations. Nat Genet (2017) 49(4):613–7. doi: 10.1038/ng.3815 PMC5373987 28288113

